# Supplementary material for: Randomised controlled trials for improving health outcomes for people living with multiple long-term conditions: Protocol for a systematic review of methodological approaches, risk of bias and reporting quality
Source: PLoS One. 2025 Jun 30;20(6):e0325742. doi: 10.1371/journal.pone.0325742 (PMC12208475; doi:10.1371/journal.pone.0325742)
Supplement: S2 File — (DOCX) [file pone.0325742.s002.docx]

**S1 Search Strategies**

**Search strategy MEDLINE (Ovid)**

exp Multimorbidity/
(multimorbid* or multi-morbid*).ti,ab,kf.

(multidisease* or multi-disease* or multicondition* or polymorbid*).ti,ab,kf.  
(multiple chronic condition* or multiple chronic disease* or multiple chronic ill*).ti,ab,kf.
(multiple long-term condition* or multiple long term condition*).ti,ab,kf.
((multiple or multi) adj (morbid* or ill* or disease* or condition* or syndrom* or disorder*)).ti,ab,kf.
exp randomized controlled trial/
controlled clinical trial.pt.
randomized.ab.
placebo.ab.
clinical trials as topic/
randomly.ab.
trial.ti.
1 or 2 or 3 or 4 or 5 or 6
7 or 8 or 9 or 10 or 11 or 12 or 13
14 and 15
exp animals/ not humans/
16 not 17
limit 18 to (english language and yr="1999-Current")

**Search strategy CINAHL (EBSCOHost)**

( (MH Multimorbidity+) OR ((TI multimorbid* OR AB multimorbid* OR SU multimorbid*) OR (TI multi-morbid* OR AB multi-morbid* OR SU multi-morbid*)) OR ((TI multidisease* OR AB multidisease* OR SU multidisease*) OR (TI multi-disease* OR AB multi-disease* OR SU multi-disease*) OR (TI multicondition* OR AB multicondition* OR SU multicondition*) OR (TI polymorbid* OR AB polymorbid* OR SU polymorbid*))OR ((TI "multiple chronic condition*" OR AB "multiple chronic condition*" OR SU "multiple chronic condition*") OR (TI "multiple chronic disease*" OR AB "multiple chronic disease*" OR SU "multiple chronic disease*") OR (TI "multiple chronic ill*" OR AB "multiple chronic ill*" OR SU "multiple chronic ill*")) OR (multiple OR multi ) W1 (morbid* OR ill* OR disease* OR condition* OR syndrom* OR disorder* ) OR ((TI "multiple long-term condition*" OR AB "multiple long-term condition*" OR SU "multiple long-term condition*") OR (TI "multiple long term condition*" OR AB "multiple long term condition*" OR SU "multiple long term condition*")) ) AND ( ((MH "Experimental Studies+") OR (MH "Multicenter Studies") OR (MH "Random Sample+") OR (MH "Placebos") OR (MH "Control (Research)+") OR (MH "Crossover Design") OR ((TI random* OR AB random*) OR (TI sham OR AB sham) OR (TI placebo* OR AB placebo*)) OR (((TI singl* OR AB singl*) OR (TI doubl* OR AB doubl*)) W1 ((TI blind* OR AB blind*) OR (TI dumm* OR AB dumm*) OR (TI mask* OR AB mask*))) OR (((TI tripl* OR AB tripl*) OR (TI trebl* OR AB trebl*)) W1 ((TI blind* OR AB blind*) OR (TI dumm* OR AB dumm*) OR (TI mask* OR AB mask*))) OR ((TI control* OR AB control*) N3 ((TI study OR AB study) OR (TI studies OR AB studies) OR (TI trial* OR AB trial*) OR (TI group* OR AB group*))) OR ((TI clinical OR AB clinical) N3 ((TI study OR AB study) OR (TI studies OR AB studies) OR (TI trial* OR AB trial*))) OR ((TI Nonrandom* OR AB Nonrandom*) OR (TI "non random*" OR AB "non random*") OR (TI "non-random*" OR AB "non-random*") OR (TI "quasi-random*" OR AB "quasi-random*") OR (TI quasirandom* OR AB quasirandom*)) OR ((TI phase OR AB phase) N6 ((TI study OR AB study) OR (TI studies OR AB studies) OR (TI trial* OR AB trial*))) OR (((TI crossover OR AB crossover) OR (TI "cross-over" OR AB "cross-over")) N3 ((TI study OR AB study) OR (TI studies OR AB studies) OR (TI trial* OR AB trial*))) OR (((TI multicent* OR AB multicent*) OR (TI "multi-cent*" OR AB "multi-cent*")) N3 ((TI study OR AB study) OR (TI studies OR AB studies) OR (TI trial* OR AB trial*))) OR (TI allocated OR AB allocated) OR (((TI "open label" OR AB "open label") OR (TI "open-label" OR AB "open-label")) N5 ((TI study OR AB study) OR (TI studies OR AB studies) OR (TI trial* OR AB trial*))) OR (((TI equivalence OR AB equivalence) OR (TI superiority OR AB superiority) OR (TI "non-inferiority" OR AB "non-inferiority") OR (TI noninferiority OR AB noninferiority)) N3 ((TI study OR AB study) OR (TI studies OR AB studies) OR (TI trial* OR AB trial*))) OR ((TI "pragmatic study" OR AB "pragmatic study") OR (TI "pragmatic studies" OR AB "pragmatic studies")) OR (((TI pragmatic OR AB pragmatic) OR (TI practical OR AB practical)) N3 (TI trial* OR AB trial*)) OR (((TI quasiexperimental OR AB quasiexperimental) OR (TI "quasi-experimental" OR AB "quasi-experimental")) N3 ((TI study OR AB study) OR (TI studies OR AB studies) OR (TI trial* OR AB trial*))) OR (TI trial)) NOT ((MH animals+) NOT (MH humans)) ) AND LA English AND DT 19990101-20241231

**Search strategy Scopus**

INDEXTERMS ( multimorbidity ) OR TITLE-ABS-KEY ( multimorbid* OR multi-morbid* ) OR TITLE-ABS-KEY ( multidisease* OR multi-disease* OR multicondition* OR polymorbid* ) OR TITLE-ABS-KEY ( "multiple chronic condition*" OR "multiple chronic disease*" OR "multiple chronic ill*" ) OR TITLE-ABS-KEY ( "multiple long-term condition*" OR "multiple long term condition*" ) AND ( INDEXTERMS ( "randomized controlled trial" ) OR DOCTYPE ( "controlled clinical trial" ) OR ABS ( randomized ) OR ABS ( placebo ) OR INDEXTERMS ( "clinical trials as topic" ) OR ABS ( randomly ) OR TITLE ( trial ) ) AND PUBYEAR > 1998 AND PUBYEAR < 2025 AND ( LIMIT-TO ( LANGUAGE , "English" ) ) AND NOT (INDEXTERMS(animals) NOT INDEXTERMS(humans))

**Search strategy CENTRAL (Cochrane Library)**

#1 [mh Multimorbidity]

#2 (multimorbid*:ti,ab,kw OR multi-morbid*:ti,ab,kw)

#3 (multidisease*:ti,ab,kw OR multi-disease*:ti,ab,kw OR multicondition*:ti,ab,kw OR polymorbid*:ti,ab,kw )

#4 (("multiple chronic" NEXT condition*):ti,ab,kw OR ("multiple chronic" NEXT disease*):ti,ab,kw OR ("multiple chronic" NEXT ill*):ti,ab,kw)

#5 (multiple OR multi ) NEXT (morbid* OR ill* OR disease* OR condition* OR syndrom* OR disorder* )

#6 (("multiple long-term" NEXT condition*):ti,ab,kw OR ("multiple long term" NEXT condition*):ti,ab,kw)

#7 #1 OR #2 OR #3 OR #4 OR #5 OR #6

#8 [mh animals] NOT [mh ^humans]

#9 #7 NOT #8 with Publication Year from 1999 to 2024, in Trials
