# Supplementary material for: Randomised controlled trials for improving health outcomes for people living with multiple long-term conditions: Protocol for a systematic review of methodological approaches, risk of bias and reporting quality
Source: PLoS One. 2025 Jun 30;20(6):e0325742. doi: 10.1371/journal.pone.0325742 (PMC12208475; doi:10.1371/journal.pone.0325742)
Supplement: Attachment — Submitted filename: PLOSONE_Multimorbidity_SRprotocol_20241202.docx [file pone.0325742.s003.docx]

PONE-D-24-47106

Randomised controlled trials for improving health outcomes for people living with multiple long-term conditions: protocol for a systematic review of methodological approaches, risk of bias and reporting quality.

Thank you for the opportunity to review this paper. I think this paper is worth publishing following some updates, especially of the methods section. Please see my comments below:

**General comments**

1. Please remove all references to “we” in the text. For example, on page 4, “Strengths and limitations”, “A comprehensive review of all randomised trials testing interventions to improve health outcomes for people living with multiple long-term conditions to assess their methodological approaches was conducted”.
2. Cross-check the paper regarding full stops, grammar and general sentence structure and flow.

**Introduction**

3. Page 5, line 76 “National research priorities include improving outcomes for people with MLTCs (1, 14). Despite more people living with MLTCs than single conditions, current care and research are largely single condition focused (15)”

Please clarify whose national research priorities you’re referring to. Is this also a global issue?

4. Page 5, line 83 “People with MLTCs have specific needs beyond those who have single conditions.” Consider adding a reference to support this.
5. Page 5, line 88. “Of the top 10 James Lind Alliance….” Suggest you explain what this group is and how this is relevant.

**Methods and Analysis**

6. Page 7, line 109, “Based on the conditions identified by Ho et al. (25). The study is expected to commence in November 2024, with searching, selection and data extraction expected to be completed by February 2025. It is expected that the results from the review will be available by the end of 2025.” It was not clear what the conditions identified by Ho are; please clarify or reword these sentences.
7. Page 7, line 114 “We will search the following electronic databases: Ovid MEDLINE, Scopus, CINAHL and Cochrane Library from January 1999 to identify relevant trials.” As per point 8 of my feedback, you state the search will commence in November 2024. Please update the search date to include an end date.
8. Page 7, line 118, “A copy of the Ovid MEDLINE search can be viewed in Supplementary Materials.” Please provide copies of electronic search strategies for all databases in the materials.
9. Page 7, lines 122-6, “We do recognise that the use of these terms has evolved over time and therefore, to make sure we do not exclude any relevant trials in MLTCs, we will screen the reference lists for selected studies and the previous reviews conducted in this area will also be screened for other potentially relevant publications (16, 22-24, 27). I suggest revising this sentence so it's shorter or split into 2 sentences.

Do you mean you will complete backward and forward citation screening?

Suggest text like this: Backward and forward citation tracking of included articles, that is, checking reference lists and searching for article citations, respectively, will be conducted in Web of Science and Google Scholar for additional literature unidentified by the search (minimising selection bias).

10. Page 8, line 126, “We will also search the comprehensive database of the International Research Community in Multimorbidity (28).” Please add this database to the methods in the abstract.
11. Page 8, line 141 “patient reported outcomes (e.g. HRQoL and measurement of physical or mental functioning) (30).” Are you including quality-of-life patient-reported outcomes? If so, please add.
12. Page 8, line 143, “We will only include studies published as full text articles in English.” Why are you only limiting to English papers? You could translate in Google translate.
13. Page 9, line 156, study selection “All eligible studies from the literature search will be imported into Covidence (app.covidence.org/) where duplicates will be removed.” Please add a reference for Covidence.
14. Page 9, line 197, onwards, I would separate out quality assessment and have this as its own section before you talk about data extraction as this is its own process. Regarding the quality assessment tools, I think you should specify the tools to be used, perhaps reference the EQUATOR network and provide examples [Experimental studies | Study Designs | EQUATOR Network](https://www.equator-network.org/?post_type=eq_guidelines&eq_guidelines_study_design=experimental-studies&eq_guidelines_clinical_specialty=0&eq_guidelines_report_section=0&s=)
15. Page 9, line 197, “The risk of bias of the included studies will be assessed using the Cochrane Risk of Bias (RoB) tool (31). This tool assesses bias across five domains, and overall, each is rated as either low, high or some concerns in terms of the risk of bias.”

I would list the Cochrane ROB five domains it may orientate and inform the reader.

How will the quality assessment be completed? i.e. independently by two reviewers?

How will the quality assessments be presented? i.e. Tabulated in the summary of findings table

16. Page 11, line 207 onwards. Regarding data synthesis. Have you considered if and how the quality appraisal may affect the main results? Consider adding a statement to that effect.
17. Page 12, line 228 onwards. Have you considered how else you will disseminate results other than peer-reviewed publications? For example, collaborators, advocacy organisations, social media etc.

**Discussion**

Generally, I find the discussion section to be an unusual inclusion in a protocol paper. However, perhaps this is journal-centric. I would usually have a discussion section in my SR paper following the results. If this section remains, here are some comments for your consideration.

18. Page 13, lines 235-7, states: “This comprehensive review of the methodological approach of trials conducted to inform the care of those living with MLTCs is in response to national and international calls for more research in this important growing clinical area.” Yet in the introduction, there appears to be more emphasis on national (perhaps UK-centric) issues. Please add a reference and align the focus across the paper.
19. Page 13, lines 240-245 “Trial advancement was also a feature of the National Institute of Health and Care Research (NIHR) strategic framework for MLTCs, with methodologies being identified as one of the strategic priority areas. The NIHR has specifically noted the need for outcome frameworks and ensuring representation within MLTC trials. This review will benchmark current methodological approaches in MLTC trials, highlighting opportunities for improvement.

Please reference these statements. Is this only NIHR centric or have other organisations highlighted these issues?

20. Page 13, lines 246-258. The text below would be better suited in the introduction as it justifies the methods, rationale, and focus of the review.

“Although previous reviews have been conducted in this area, they have been focussed on

intervention effectiveness both generically (16) and focussed on specific types of

intervention (such as models of care (33), behaviour change interventions (24), medication

249 adherence (34)), or on specific methodological issues such as ethnic representation (22) or MLTC subgroups, such as those with frailty plus MLTC (27). Unlike these previous studies

we will not include terms related to comorbidity in our searches. This was a pragmatic

decision to limit the number of studies needed to be screened and given that our interest is

in trials of interventions aimed at those with MLTCs in the broadest sense and not those

living with an index condition plus another (i.e., comorbidity). Any studies which have used

terms related to comorbidity to describe a MLTC population will be picked up through

screening the references of previous systematic reviews in this area. Given the terms

multimorbidity and MLTC are becoming the more standard way over time to describe this

patient group, we hope this will have a limited impact on more recent studies.”
