# Supplementary material for: Randomised controlled trials for improving health outcomes for people living with multiple long-term conditions: Protocol for a systematic review of methodological approaches, risk of bias and reporting quality
Source: PLoS One. 2025 Jun 30;20(6):e0325742. doi: 10.1371/journal.pone.0325742 (PMC12208475; doi:10.1371/journal.pone.0325742)
Supplement: Attachment — Submitted filename: Response to reviewers.docx [file pone.0325742.s005.docx]

**Response to reviewer’s comments**

***We thank the reviewer for their comments and insight, we have taken onboard these suggestions and updated the manuscript. Below we have addressed each comment in turn. Where required we have also made the methodology more explicit.***

**General comments**

**Comment:** Please remove all references to “we” in the text. For example, on page 4, “Strengths and limitations”, “A comprehensive review of all randomised trials testing interventions to improve health outcomes for people living with multiple long-term conditions to assess their methodological approaches was conducted”.

**Response:** All references to ‘we’ have been removed from the manuscript.

**Comment:** Cross-check the paper regarding full stops, grammar and general sentence structure and flow.

**Response:** The grammar and sentence structure and flow has been thoroughly reviewed.

**Introduction**

**Comment:** Page 5, line 76 “National research priorities include improving outcomes for people with MLTCs (1, 14). Despite more people living with MLTCs than single conditions, current care and research are largely single condition focused (15)” Please clarify whose national research priorities you’re referring to. Is this also a global issue?

**Response:** this has been clarified.

**Comment:** Page 5, line 83 “People with MLTCs have specific needs beyond those who have single conditions.” Consider adding a reference to support this.

**Response:** A reference has been added.

**Comment:** Page 5, line 88. “Of the top 10 James Lind Alliance….” Suggest you explain what this group is and how this is relevant.

**Response:** This has been clarified.

**Methods and Analysis**

**Comment:** Page 7, line 109, “Based on the conditions identified by Ho et al. (25). The study is expected to commence in November 2024, with searching, selection and data extraction expected to be completed by February 2025. It is expected that the results from the review will be available by the end of 2025.” It was not clear what the conditions identified by Ho are; please clarify or reword these sentences.

**Response:** We have removed this sentence as we agreed it is not clear.

**Comment:** Page 7, line 114 “We will search the following electronic databases: Ovid MEDLINE, Scopus, CINAHL and Cochrane Library from January 1999 to identify relevant trials.” As per point 8 of my feedback, you state the search will commence in November 2024. Please update the search date to include an end date.

**Response:** This has been clarified.

**Comment:** Page 7, line 118, “A copy of the Ovid MEDLINE search can be viewed in Supplementary Materials.” Please provide copies of electronic search strategies for all databases in the materials.

**Response:** As per the PRISMA-P guidelines only one search strategy is required. We have now added the others.

**Comment:** Page 7, lines 122-6, “We do recognise that the use of these terms has evolved over time and therefore, to make sure we do not exclude any relevant trials in MLTCs, we will screen the reference lists for selected studies and the previous reviews conducted in this area will also be screened for other potentially relevant publications (16, 22-24, 27). I suggest revising this sentence so it's shorter or split into 2 sentences.

Do you mean you will complete backward and forward citation screening?

Suggest text like this: Backward and forward citation tracking of included articles, that is, checking reference lists and searching for article citations, respectively, will be conducted in Web of Science and Google Scholar for additional literature unidentified by the search (minimising selection bias).

**Response:** We will not be conducted forward citation searching. We have amended the text to reflect that we will be conducting backwards citation searching and broken down the sentences.

**Comment:** Page 8, line 126, “We will also search the comprehensive database of the International Research Community in Multimorbidity (28).” Please add this database to the methods in the abstract.

**Response:** We have added this as suggested.

**Comment:** Page 8, line 141 “patient reported outcomes (e.g. HRQoL and measurement of physical or mental functioning) (30).” Are you including quality-of-life patient-reported outcomes? If so, please add.

**Response:** This has been amended to include QoL.

**Comment:** Page 8, line 143, “We will only include studies published as full text articles in English.” Why are you only limiting to English papers? You could translate in Google translate.

**Response:** We are not comfortable with using google translate in terms of the validity of the translations for complex medical terminology and potential bias. We will report how many studies are excluded due to this decision.

**Comment:** Page 9, line 156, study selection “All eligible studies from the literature search will be imported into Covidence (app.covidence.org/) where duplicates will be removed.” Please add a reference for Covidence.

**Response:** Reference added.

**Comment:** Page 9, line 197, onwards, I would separate out quality assessment and have this as its own section before you talk about data extraction as this is its own process. Regarding the quality assessment tools, I think you should specify the tools to be used, perhaps reference the EQUATOR network and provide examples [Experimental studies | Study Designs | EQUATOR Network](https://www.equator-network.org/?post_type=eq_guidelines&eq_guidelines_study_design=experimental-studies&eq_guidelines_clinical_specialty=0&eq_guidelines_report_section=0&s=)

**Response:** We have not carried out a quality assessment beyond assessing the risk of bias. We have amended this section to reflect this.

**Comment:** Page 9, line 197, “The risk of bias of the included studies will be assessed using the Cochrane Risk of Bias (RoB) tool (31). This tool assesses bias across five domains, and overall, each is rated as either low, high or some concerns in terms of the risk of bias.”

I would list the Cochrane ROB five domains it may orientate and inform the reader.

**Response:** We have added these.

**Comment:** How will the quality assessment be completed? i.e. independently by two reviewers?

**Response:** We are not completing a quality assessment, all data extracted including the risk of bias will be extracted independently by two reviewers. We have made this clearer in the text.

**Comment:** How will the quality assessments be presented? i.e. Tabulated in the summary of findings table

**Response:** We are not completing a quality assessment, we have made the synthesis section clearer in terms of how the risk of bias and CONSORT data will be displayed.

**Comment:** Page 11, line 207 onwards. Regarding data synthesis. Have you considered if and how the quality appraisal may affect the main results? Consider adding a statement to that effect.

**Response:** We have added a statement as suggested.

**Comment:** Page 12, line 228 onwards. Have you considered how else you will disseminate results other than peer-reviewed publications? For example, collaborators, advocacy organisations, social media etc.

**Response:** We will be sharing the results via webinars. The results will also be shared via social media and we have added this as suggested.

**Discussion**

**Comment:** Generally, I find the discussion section to be an unusual inclusion in a protocol paper. However, perhaps this is journal-centric. I would usually have a discussion section in my SR paper following the results. If this section remains, here are some comments for your consideration.

**Response:** We have followed the journal guidelines regarding manuscript sections.

**Comment:** Page 13, lines 235-7, states: “This comprehensive review of the methodological approach of trials conducted to inform the care of those living with MLTCs is in response to national and international calls for more research in this important growing clinical area.” Yet in the introduction, there appears to be more emphasis on national (perhaps UK-centric) issues. Please add a reference and align the focus across the paper.

**Response:** We have addressed the introduction regarding global interest and expanded this section to specifically cover this.

**Comment:** Page 13, lines 240-245 “Trial advancement was also a feature of the National Institute of Health and Care Research (NIHR) strategic framework for MLTCs, with methodologies being identified as one of the strategic priority areas. The NIHR has specifically noted the need for outcome frameworks and ensuring representation within MLTC trials. This review will benchmark current methodological approaches in MLTC trials, highlighting opportunities for improvement.

Please reference these statements. Is this only NIHR centric or have other organisations highlighted these issues?

**Response:** We have referenced these statements and updated this section to include the international context.

**Comment:** Page 13, lines 246-258. The text below would be better suited in the introduction as it justifies the methods, rationale, and focus of the review.

“Although previous reviews have been conducted in this area, they have been focussed on intervention effectiveness both generically (16) and focussed on specific types of intervention (such as models of care (33), behaviour change interventions (24), medication 249 adherence (34)), or on specific methodological issues such as ethnic representation (22) or MLTC subgroups, such as those with frailty plus MLTC (27). Unlike these previous studies we will not include terms related to comorbidity in our searches. This was a pragmatic decision to limit the number of studies needed to be screened and given that our interest is in trials of interventions aimed at those with MLTCs in the broadest sense and not those living with an index condition plus another (i.e., comorbidity). Any studies which have used terms related to comorbidity to describe a MLTC population will be picked up through screening the references of previous systematic reviews in this area. Given the terms multimorbidity and MLTC are becoming the more standard way over time to describe this patient group, we hope this will have a limited impact on more recent studies.”

**Response:** We thank the reviewer for this comment, we have moved the first part of this section to the introduction.
