# Supplementary material for: Randomised controlled trials for improving health outcomes for people living with multiple long-term conditions: Protocol for a systematic review of methodological approaches, risk of bias and reporting quality
Source: PLoS One. 2025 Jun 30;20(6):e0325742. doi: 10.1371/journal.pone.0325742 (PMC12208475; doi:10.1371/journal.pone.0325742)
Supplement: Attachment — Submitted filename: Response_to_reviewers_auresp_2.docx [file pone.0325742.s006.docx]

**Response to reviewers’ comments**

**Reviewer #2**

**Comment:** Thank you for pursuing research into this important topic.

**Response:** We thank the reviewer for this positive comment.

**Comment:** Given your interest in trials specifically, I would assume you are searching Cochrane Central rather than the entire Cochrane Library - please clarify the abstract. I also find it odd to identify the interface in the abstract for only one of the databases; would suggest deferring that detail to the text.

**Response:** We have corrected and made consistent the list of databases used.

**Comment:** In defining the topic, you use the terminology "long duration" - what does this mean specifically? How long?

**Response:** We have added a definition of long-duration to the introduction.

**Comment:** Is the "two-thirds of adults aged over 65 will be affected" estimate specific to England, as in the preceding clause, or globally? Suggest clarifying.

**Response:** We have clarified that this estimate is specific to England.

**Comment:** "Despite more people living with MLTCs than single conditions" - what is the source for this claim? I'm not seeing it in the following reference.

**Response:** We apologise for this mistake, we have corrected the text.

**Comment:** The manuscript would benefit from a thorough copy-editing for clarity and flow.

**Response:** We have proof read the article and made improvements to the grammar and readability.

**Comment:** The body of the manuscript should identify specifically the interfaces through which each database is being searched, and see comment above about Cochrane Library.

**Response:** We have now identified both the databases and the interfaces.

**Comment:** "The start date of January 1999 was chosen, as this is when the first trial in MLTCs was reported in a previous systematic review". The review referenced was limited to those taking place in primary care and community settings, which does not appear to be the case for your review - there may well be earlier trials in other contexts.

**Response:**

**Comment:** A rationale should be provided for limiting results to English.

**Response:** We have now included the rationale.

**Comment:** Was consideration given to searching the grey literature? Its absence should be noted as a limitation.

**Response:** We have included this as a limitation in the discussion.

**Comment:** What is the rationale for excluding secondary/subgroup analyses?

**Response:** The rational has been added.

**Comment:** The Scopus search strategy appears to be missing parentheses around the multimorbidity concept. Also why not use the same proximity search strings as for the other databases?

**Response:** The search strategy has been checked by our librarian. The proximity settings for this database were changed to limit the size of the results, this change gave a reasonable number of results but was still able to identify the core papers included in other reviews.

**Comment:** The Medline search string presented appears to be missing several lines - the final line attempts to limit line 21, but the strategy before that point only has 17 lines.

**Response:** The medline search has been corrected.

**Comment:** It appears that some literature uses the alternative term "polymorbidity" - this should be included in the search.

**Response:** We thank the reviewer for this suggestion, we have now included this term in the search strategies.

**Reviewer #3:** 

**Comment:** It was interesting reading your revised protocol. The addressed topic is fundamental to improve the quality of life of people living with multiple long-term conditions. Your revised manuscript addressed almost all the previous reviewer comments which align completely with my feedback.

**Response:** We thank the reviewer for their positive comment.

**Comment:** It is acceptable to limit the inclusion of studies to English. However, it would be recommended to justify this language restriction as long as you are not searching for studies conducted only in the UK.

**Response:** We are not limiting inclusion by the country the study was conducted in, this has been added.

**Comment:** You did not separate the risk of bias into a different subsection, which is recommended for better structure and readability.

**Response:** We have now separated the risk of bias into a subsection.

**Comment:** Lastly, it is recommended to fill out the PRISMA-P checklist and add it as a supplementary

file.

**Response:** We have completed a PRISMA-P checklist.

**Comment:** Otherwise, your protocol is to be accepted and look forward toward reading your final results.

**Response:** We thank the reviewer for their support.

**Reviewer #4**

**Comment:** The analysis of methodological aspects of RCTs on multimorbidity is a valuable research aim. However, the language and structure of this protocol require significant improvements.

**Response:** We thank the reviewer for their positive comment and have worked to improve the language and structure.

**Comment:** The sentence: "In line with the reviews by Smith et al, trials with an index condition ... will be excluded." needs further clarification.

Specifically, if a trial includes patients with diabetes (DM) and hypertension (HTN), how will it be classified?
Does inclusion depend on how the study defines the patient population? For example:
If a study states, “we evaluated diabetic patients with hypertension,” does this make diabetes the index condition, leading to exclusion?
If it states, “we evaluated hypertensive patients with diabetes,” does this make hypertension the index condition, leading to exclusion?
If it simply states “we evaluated patients with diabetes and hypertension,” does this qualify as a multimorbidity study?!

or you would exclude studies on any specific combination of disorders and only include those RCT with multimorbidity? this part of selection is not clear.

These criteria must be explicitly defined so that others can replicate the systematic review and consistently determine which trials are included.
**Response:** We have clarified the methods and added that trials of comorbidity will be excluded (i.e. studies which focus on 2 conditions specifically).

**Comment:** The authors justify using January 1999 as the starting point, stating that “this is when the first trial in MLTCs was reported in a previous systematic review.” However, the following reference suggests relevant trials exist before 1999: <https://doi.org/10.7326/0003-4819-126-12-199706150-00004>.
Given this, it may be more appropriate not to limit the search by a starting year unless a strong rationale is provided.
**Response:** The example study does not meet the inclusion criteria for the review, as diabetes is an index condition with hypertension as a comorbidity. We want the review to reflect current practice in terms of the design and analysis of trials for MLTC and therefore we believe this start date represents a pragmatic choice and has been used by other studies in this area.

**Comment:** The manuscript contains several grammatical errors. For instance, in the first two sentences of the introduction, the authors inconsistently use “is” and “are” when referring to MLTCs.
Careful proofreading is required to ensure grammatical consistency and readability.
**Response:** We have proof read the article and made improvements to the grammar and readability.

**Comment:** If the first sentence is a direct quotation, the source must be explicitly stated.
Additionally, citing four references for a simple statement is excessive and unprofessional. The authors should ensure that only the most relevant sources are cited.

**Response:** Thank you for your comment, given the definition is repeated in the methods we have removed the direct quotation from the introduction.

**Reviewer #5**

**Comment:** The edited manuscript has been checked over as well as the comments and responses. Seems fine after responding to all the requested comments from the reviewers.

**Response:** Thank you for your positive comment.
