# Supplementary material for: Randomised controlled trials for improving health outcomes for people living with multiple long-term conditions: Protocol for a systematic review of methodological approaches, risk of bias and reporting quality
Source: PLoS One. 2025 Jun 30;20(6):e0325742. doi: 10.1371/journal.pone.0325742 (PMC12208475; doi:10.1371/journal.pone.0325742)
Supplement: Attachment — Submitted filename: Response_to_reviewers_auresp_3.docx [file pone.0325742.s007.docx]

**Response to reviewers**

Comment: I would like authors to reconsider some points regarding the reference section. some references have a link to download a file but when we click on it, it leads to empty web page.

Response: We have updated the references, paying specific attention to those of websites/blogs.

Comment: Do you mean all articles in this website page are this single reference?

Response: Yes, we have updated the text to make this clearer.

Comment: “Not doing a meta-analysis due to the research question" needs some more clarification. You are including RCT studies and your PICO is in quantitative format. This means the results should be able to be running a meta-analysis. The reason does not appear to be valid.

Response: The research question for this review does not consider effectiveness and therefore conducting a meta-analysis is not required to answer the research question. We are interested in the current approaches to design and analysis for MLTC trials. The results from this study will feed into a Delphi study which will prioritise research uncertainties in this area. This type of systematic review is not uncommon in methodological research and can be highly cited as useful for driving future research in this area. A few examples are given below

Eldridge SM, Ashby D, Feder GS, Rudnicka AR, Ukoumunne OC. Lessons for cluster randomized trials in the twenty-first century: a systematic review of trials in primary care. Clin Trials. 2004 Feb;1(1):80-90. doi: 10.1191/1740774504cn006rr. PMID: 16281464.

Varghese E, Briola A, Kennel T, Pooley A, Parker RA. A systematic review of stepped wedge cluster randomized trials in high impact journals: assessing the design, rationale, and analysis. J Clin Epidemiol. 2025 Feb;178:111622. doi: 10.1016/j.jclinepi.2024.111622. Epub 2024 Dec 2. PMID: 39631553.

Comment: I retaliate what the second and fourth reviewers said, that a thorough grammatical check should be carried out before submitting the manuscript. (Check out line 197 and 198 and rectify them to make grammatical sense).

Response: We have completed a thorough review of the grammar, paying particular attention to the lines suggested by the reviewer.
